# Severe neutrophilic leukocytosis as a progression marker in granulocyte colony‐stimulating factor‐producing squamous cell carcinoma of the esophagus

**DOI:** 10.1002/ccr3.908

**Published:** 2017-03-31

**Authors:** Shun Yamaguchi, Kengo Kanetaka, Shinichiro Kobayashi, Yasuhiro Nagata, Naoe Kinosita, Junya Fukuoka, Shunsuke Murakami, Fumihiko Fujita, Mitsuhisa Takatsuki, Susumu Eguchi

**Affiliations:** ^1^ Department of SurgeryNagasaki University Graduate School of Biomedical SciencesNagasakiJapan; ^2^ Center for Comprehensive Community Care EducationNagasaki University Graduate School of Biomedical SciencesNagasakiJapan; ^3^ Department of PathologyNagasaki University HospitalNagasakiJapan

**Keywords:** Esophagus, G‐CSF, squamous cell carcinoma

## Abstract

There are very few reports of esophageal carcinoma producing granulocyte colony‐stimulating factor (G‐CSF). G‐CSF‐producing esophageal squamous cell carcinoma is an extremely aggressive carcinoma. Leukocyte counts, neutrophil counts, and serum C‐reactive protein levels may be markers of its progression.

## Background

Patients with malignant neoplasms may show various levels of leukocytosis because of accompanying bacterial infections, such as aspiration pneumonia in esophageal cancer and obstructive colitis in advanced colon cancer 1. The presence of cancer itself can lead to the development of leukocytosis as a reaction against the neoplasm. However, extreme leukocytosis is rarely encountered without a bacterial infection. In 1952, Hughes et al. first reported a case of carcinomatosis in a patient who developed extreme leukocytosis via the production of granulocyte colony‐stimulating factor (G‐CSF), which originated from the tumor 2. Although G‐CSF‐producing cancer has been reported in various organs, including the lung, urinary tract, stomach, and pancreas 3, 4, 5, 6, 7, reports of G‐CSF‐producing esophageal carcinoma are rare. As G‐CSF‐producing tumors are reported to be a rapidly growing phenotype that is associated with a poor prognosis, it is crucial to correctly diagnose and apply an effective treatment as early as possible 2. Here, we report a case of G‐CSF‐producing esophageal squamous cell carcinoma that spread rapidly to the liver within 1 month after its initial presentation. We also discuss the prognosis and treatment of G‐CSF‐producing esophageal cancer via a review of the literature.

## Case Presentation

**Figure 1:**
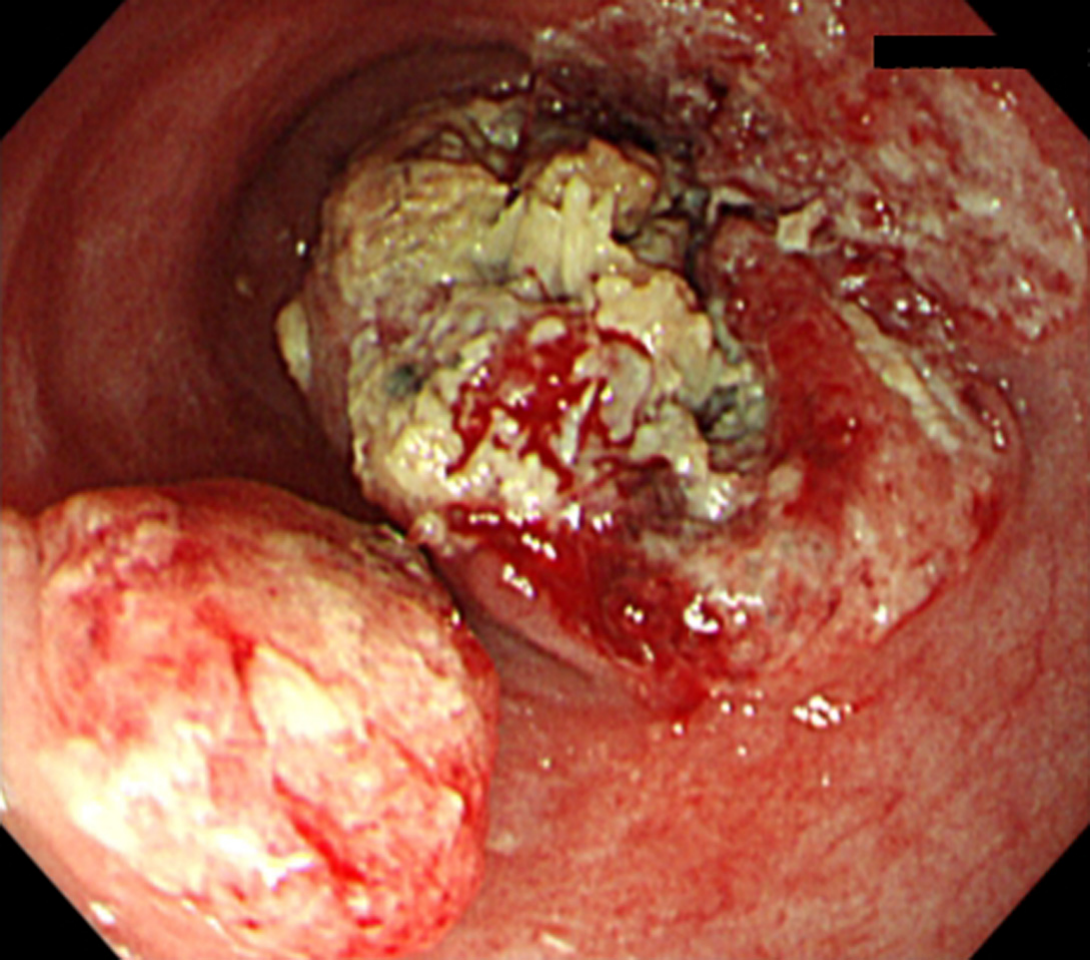
Esophagoscopy. A large neoplasm was detected in the lower esophagus.

**Figure 2:**
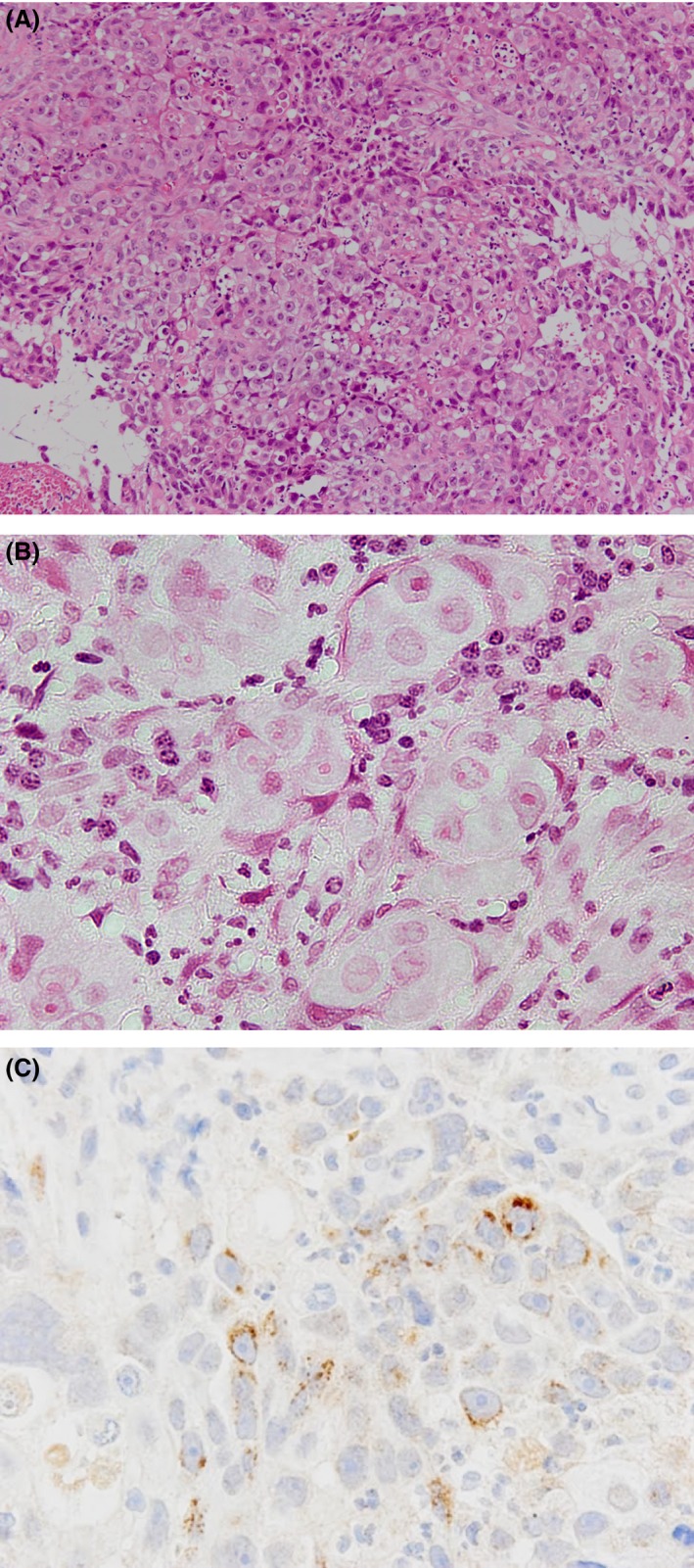
The histopathological findings. (A) The specimen was identified as a poorly differentiated squamous cell carcinoma (hematoxylin and eosin staining). (B) Neutrophil infiltration was observed around the malignant cells (hematoxylin and eosin staining). (C) The immunohistochemical staining of biopsied specimens with anti‐G‐CSF antibody showing many positive cancer cells.

**Figure 3:**
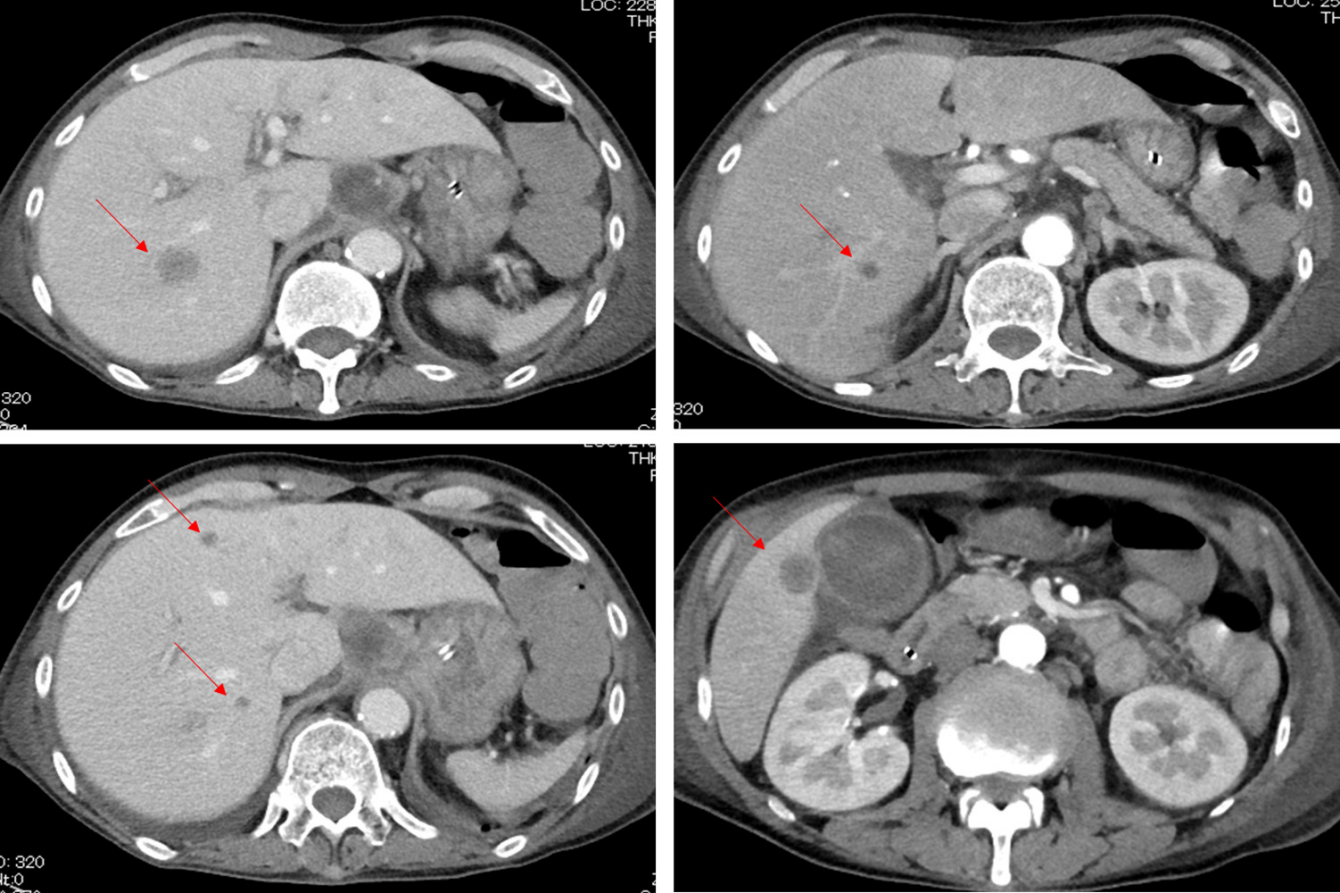
Contrast CT imaging. The arrows indicate the metastatic liver lesion.

**Figure 4:**
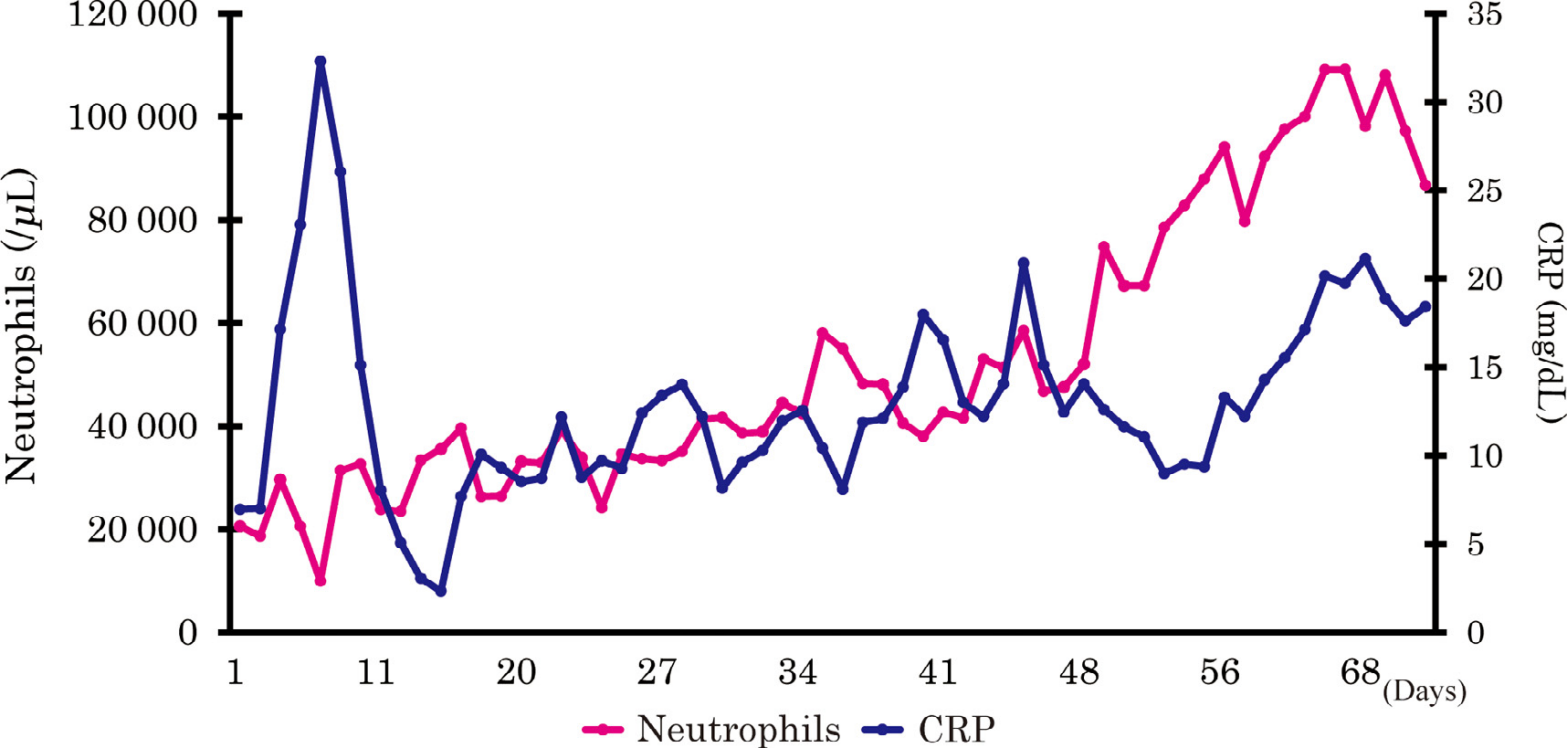
The changes in neutrophil count and serum C‐reactive protein (CRP) level. The neutrophil count and serum CRP level gradually increased as the tumor progressed.

The patient, a Japanese man in his 60s, presented at our hospital with dysphagia. He also presented with general fatigue and severe weight loss. An upper gastrointestinal endoscopic examination revealed a full‐circumferential, type‐three neoplasm in the lower esophagus; the scope could not be advanced past the tumor (Fig. 1). A biopsy was performed, and the pathological examination revealed a poorly differentiated squamous cell carcinoma with massive neutrophil infiltration (Fig. 2). The chest CT findings showed a thickened esophageal wall in the middle‐lower esophagus, which seemed to be in accordance with the tumor that was found during the endoscopic examination. There were several swollen lymph nodes surrounding the tumor; however, no metastatic nodules were found in distant organs, including the lungs and liver. The clinical diagnosis, according to the seventh edition of the Union for International Cancer Control, was T3N1 M0 c‐Stage III esophageal cancer 8. A radical operation with perioperative chemotherapy was planned; however, the patient's general condition deteriorated due to aspiration pneumonia from the esophageal stenosis. The intravenous administration of antibiotics and the insertion of a stent through the tumor to prevent the pooling of salivary juice in the esophagus seemed to alleviate the patient's aspiration pneumonia in terms of his fever and physical examination results. A chest CT showed no signs of inflammation in the lungs; however, despite the improvement in his condition, his white blood cell (WBC) count markedly increased to 25,100 cells/*μ*L, and his neutrophil count was also elevated at 20,600 cells/*μ*L. This unreasonable elevation of his blood counts prompted the analysis of his serum G‐CSF level, which was elevated (292 pg/mL; normal range, 5.78–27.5 pg/mL), and an immunohistochemical examination of the biopsy specimens also revealed that the cancer cells were G‐CSF‐positive (Fig. 2). Although his physical condition recovered 1 month after admission, his WBC and neutrophil counts increased to 55,400 and 48,200 cells/*μ*L, respectively. His serum CRP level was elevated at 10 mg/dL. An abdominal CT scan showed that multiple liver metastases had developed (Fig. 3). Despite the provision of best supportive care, including administration of an antipyretic to treat the high fever, his WBC and neutrophil counts gradually increased to 112,600 and 108,100 cells/*μ*L, respectively. His serum CRP level elevated to 20 mg/dL (Fig. 4). He died 3 months after the definite diagnosis.

## Discussion

Herein, we report a case of G‐CSF‐producing esophageal squamous cell carcinoma. The WBC count, neutrophil count, and CRP continued to increase despite the alleviation of pneumonia through intravenous antibiotics and insertion of an esophageal stent. The tumor rapidly spreads to the liver within 1 month of the definitive diagnosis, and he died within 3 months of the definitive diagnosis. Thus, WBC count, neutrophil count, and serum CRP level may reflect the progression of G‐CSF‐producing esophageal squamous cell carcinoma. The neutrophil counts are especially important as a progression marker.

Granulocyte colony‐stimulating factor is a hematopoietic growth factor that regulates the production of granulocytes and macrophages 9. Several reports have mentioned the diagnostic criteria for a G‐CSF‐producing tumor, which include the following: (A) marked leukocytosis, (B) a high serum concentration of G‐CSF, (C) a decreasing leukocyte count after tumor resection, and (D) evidence of G‐CSF production in the tumor cells 10. In this case, the leukocyte count was elevated to more than 25,000 cells/*μ*L, and the production of G‐CSF from the neoplasm was confirmed by the patient's high serum levels of G‐CSF and an immunohistochemical examination. Thus, our case fulfilled parts (A, B, and D) of the diagnostic criteria.

We were able to diagnose G‐CSF‐producing squamous cell carcinoma using a small block of biopsied specimens. First, the histopathological findings of the biopsied specimens showed atypical findings, such as remarkable neutrophil infiltration surrounding the malignant cells. With suspicion of G‐CSF production, we performed an immunohistochemical analysis, which revealed numerous G‐CSF‐positive malignant cells. In contrast, Shimomura reported that it was difficult to detect G‐CSF in an immunohistological examination because G‐CSF is rapidly secreted from cancer cells after production in vitro 11. In the case of esophageal carcinosarcoma, Sasaki et al. reported that G‐CSF expression was also detected by an immunohistological examination but only in some of the carcinomatous cells 12. Thus, when biopsy specimens show remarkable neutrophil infiltration but are negative for G‐CSF, an immunohistochemical examination of biopsy specimens should be repeated to determine whether some components include G‐CSF‐positive cancer cells, which may lead to a definitive diagnosis of a G‐CSF‐producing tumor and the prompt initiation of treatment.

To the best of our knowledge, there have been only 10 reported cases of G‐CSF‐producing esophageal squamous cell carcinomas 13, 14, 15, 16, 17, 18, 19, 20, 21 (Table 1). The majority of patients were male (nine of ten cases), and the average age of the patients was 63.4 ± 14.2 years (range, 30–81 years). As reported in cases involving G‐CSF‐produ cing tumors in other organs, G‐CSF‐producing esophageal squamous cell carcinoma is a very aggressive phenotype; in three of the ten reported cases, a radical operation could not be performed due to rapid growth and spread to distant organs. The most common complaint of patients with G‐CSF‐producing esophageal cancer is dysphagia (seven of ten cases). Moreover, even if radical treatments were performed, early recurrence developed within several months in six of the seven cases. Nine of the ten patients died within 16 months after diagnosis (range: 0.5–16 months). Leukocytosis developed in all nine cases. The serum G‐CSF level was elevated in all cases.

**Table 1:** The clinical characteristics of G‐CSF‐producing esophageal cancer

Author	Year	Gender	Age	G‐CSF level (pg/mL)	WBC (/*μ*L)	Chief complaint	Treatment	Outcome
Watanabe	1999	F	81	1175	NA	Dysphagia	BSC	0.5 months, dead
Matsumoto	2000	M	66	154	22,100	Dysphagia	Resection with CRT	16 months, dead
Ichiishi	2000	M	66	180	42,500	General malaise	BSC	2 months, dead
Kato	2002	M	54	150	33,900	Abdominal pain	Chemotherapy	3 months, dead
Nakata	2006	M	56	78	16,900	Pyrexia	Resection with CRT	16 months, alive
Mimatsu	2008	M	69	113	NA	Dysphagia	Radiation	7 months, dead
Tanabe	2009	M	76	134	19,600	Dysphagia	Resection with CRT	10 months, dead
Mayanagi	2013	M	30	53.7	24,600	Dysphagia	Resection after CRT	3 months, recurrence
Shimakawa	2014	M	70	254	19,020	Dysphagia	Resection	12 months, dead
Present case	2016	M	66	292	25,100	Dysphagia	BSC	3 months, dead

WBC, white blood cell; M, male; F, female; BSC, best supportive care; NA, not available; CRT, chemoradiation therapy.

Granulocyte colony‐stimulating factor is a key regulator of neutrophil production. In addition, the correlation between G‐CSF and CRP has been reported in several conditions 22, 23. In this case, parallel increases in neutrophils and CRP were observed. The continuous increase in neutrophils and serum CRP level may reflect tumor progression.

The opportunity to administer radical treatment is often lost in patients with G‐CSF‐producing esophageal cancer due to the rapid progression of the disease. Similarly, to ordinary esophageal cancer, surgical resection with neoadjuvant therapy is considered to be the mainstay radical treatment for G‐CSF‐producing esophageal cancer. In Japan, the standard treatment strategy includes radical esophagectomy through a thoracotomy or a thoracoscopic approach with neoadjuvant chemotherapy using 5‐FU and CDDP 24. The theoretical benefit of neoadjuvant chemotherapy is the eradication of cancer cells that spill from the main tumor before definitive resection. As has been reported with neoadjuvant chemotherapy for breast cancer, the achievement of a pathological complete response in the resected specimen is indicative of both an excellent response to chemotherapy and a good postoperative prognosis 25. Mayanagi et al. reported a patient who had a recurrence of regional and distant lymph node metastases 3 months after surgery, despite neoadjuvant chemoradiotherapy that was extremely effective in achieving a pathological complete response 20. Shimakawa et al. also reported a patient with G‐CSF‐producing esophageal cancer who received preoperative chemotherapy (5‐FU, ADM, and CDDP) and whose resected specimens revealed no demonstrable cancer cells. However, liver metastasis developed within 3 months after surgery 21. In our patient, the early metastasis in the liver may imply that the tumor cells had already metastasized at the time of presentation. Therefore, it remains controversial as to whether a radical operation, combined with 5‐FU‐based chemotherapy or chemoradiotherapy, is feasible for G‐CSF‐producing esophageal cancer.

Several studies have reported that the autocrine and paracrine secretions of G‐CSF via the JAK2/STAT3 pathway confer more proliferative, invasive, and chemoresistant features to malignant cells 26. Furthermore, G‐CSF is known to promote tumor growth by stimulating angiogenesis 27. Thus, novel therapies targeting these molecular mechanisms, such as the JAK2/STAT3 pathway and angiogenesis, are expected in the future.

In conclusion, we report a case of aggressive G‐CSF‐producing esophageal squamous cell carcinoma. WBC, neutrophils, and serum CRP levels may be markers of its progression. There is currently no established therapeutic strategy for this disease, and a multidisciplinary approach will be required to overcome G‐CSF‐producing esophageal squamous cell carcinoma in the future.

## Authorship

All of the authors have read and approved the manuscript. SY and SK: collected, analyzed, and interpreted the patient disease data and edited the manuscript. SY and SK: contributed equally to the manuscript. KK: supervised the patients’ treatments and the research project. YN, FF, and MT: participated in the discussion. NK and JF: are pathologists and participated in the diagnosis of G‐CSF‐producing esophageal cancer. SM: also collected the data and participated in the case presentation. SE: approved the final submission of this manuscript.

## Consent for Publication

Informed consent for this report was obtained from the patient and his family.

## Ethics Approval

The publication of the present study was in accordance with the ethical standards of our institution.

## Conflict of Interest

The authors declare no conflicts of interest in association with the present study.
